# Can local-community-paradigm and epitopological learning enhance our understanding of how local brain connectivity is able to process, learn and memorize chronic pain?

**DOI:** 10.1007/s41109-017-0048-x

**Published:** 2017-08-30

**Authors:** Vaibhav Narula, Antonio Giuliano Zippo, Alessandro Muscoloni, Gabriele Eliseo M. Biella, Carlo Vittorio Cannistraci

**Affiliations:** 1 Biomedical Cybernetics Group, Biotechnology Center (BIOTEC), Center for Molecular and Cellular Bioengineering (CMCB), Center for Systems Biology Dresden (CSBD), Department of Physics, Technische Universität Dresden, Dresden, Germany; 2 Brain bio-inspired computatiing (BBC) lab, IRCCS Centro Neurolesi “Bonino Pulejo”, Messina, Italy; 3 Institute of Molecular Bioimaging and Physiology, Consiglio Nazionale delle Ricerche, Segrate, Milan, Italy

**Keywords:** Network topology, Topological measures, Local-community-paradigm, Brain connectivity, Pain markers, Computational neuroscience, Learning, Memory

## Abstract

The mystery behind the origin of the pain and the difficulty to propose methodologies for its quantitative characterization fascinated philosophers (and then scientists) from the dawn of our modern society. Nowadays, studying patterns of information flow in mesoscale activity of brain networks is a valuable strategy to offer answers in computational neuroscience. In this paper, complex network analysis was performed on the time-varying brain functional connectomes of a rat model of persistent peripheral neuropathic pain, obtained by means of local field potential and spike train analysis. A wide range of topological network measures (14 in total, the code is publicly released at: https://github.com/biomedical-cybernetics/topological_measures_wide_analysis) was employed to quantitatively investigate the rewiring mechanisms of the brain regions responsible for development and upkeep of pain along time, from three hours to 16 days after nerve injury. The time trend (across the days) of each network measure was correlated with a behavioural test for rat pain, and surprisingly we found that the rewiring mechanisms associated with two local topological measure, the local-community-paradigm and the power-lawness, showed very high statistical correlations (higher than 0.9, being the maximum value 1) with the behavioural test. We also disclosed clear functional connectivity patterns that emerged in association with chronic pain in the primary somatosensory cortex (S1) and ventral posterolateral (VPL) nuclei of thalamus. This study represents a pioneering attempt to exploit network science models in order to elucidate the mechanisms of brain region re-wiring and engram formations that are associated with chronic pain in mammalians. We conclude that the *local-community-paradigm* is a model of complex network organization that triggers a *local learning rule,* which seems associated to processing, learning and memorization of chronic pain in the brain functional connectivity. This rule is based exclusively on the network topology, hence was named *epitopological learning*.

**Electronic supplementary material:**

The online version of this article (doi:10.1007/s41109-017-0048-x) contains supplementary material, which is available to authorized users.

## Introduction

Life promotes changes to facilitate adaptability in environments subject to modifications. And, in the central nervous system, such dynamical adjustments are faced by means of neuroplasticity: the phenomena which enables the brain to learn by modifying its own structural and functional organization. Learning enables the brain to respond differently to the same stimulus. The retention of this difference in response is called memory. The memory can be of anything, a storage bank of information, or the protocol on how to use the information. It allows for many important life functions, for instance: gait, language, emotions or pain etc. In a general sense, the objective of this study moves around the idea of investigating how mammals process and learn pain. Specifically, we are interested in characterizing the neuroplastic correlates that distinguish acute from time-persistent (chronic) pain.

Pain pathologies can be broadly categorized into two groups: nociceptive and neuropathic. Nociceptors are the peripheral receptors that carry signals from different parts of the body corresponding to any kind of damage, e.g. tissue irritation or injury. Pain associated with the signals of nociceptors is called nociceptive pain (Cohen and Mao 2014). On the other hand, neuropathic pain is associated only with nerve injury and can be further divided into central and peripheral respectively associated with injury to nerve in central or peripheral nervous system. The intelligentsia in the field of neuroscience has come to the conclusion that different functionally specific regions of brain are responsible for distinct neuronal, bodily and behavioural processes. Research in past two decades has concluded that somatosensory cortex, thalamus and thalamo-cortical pathways are the most participating regions in processes of chronic pain (Ab Aziz and Ahmad 2006; Alshelh et al. 2016). The connectome is the network that describes the connectivity between brain regions (Cannistraci et al. 2013a). The research that investigates modifications and alterations in connectomics of affected brain regions is an interesting and novel territory of investigation. Therefore, in this paper we intend to quantitatively understand (through complex network analysis) the time-varying re-adjustments of the functional connectomes relative to rat brain regions participating in processing of peripheral neuropathic pain. This study will give us an understanding of how the functional connectomes of certain brain areas affected by and participating in peripheral neuropathy evolve as the pain states progress from acute to chronic condition. Indeed, an injury, as it heals, leaves a memory imprinting in the brain, in the form of structural and functional connectivity modifications: a neuroplasticity trace which can be temporary or persistent. Such alterations, if registered in the brain for long periods of time (from weeks to months), lead to remembrance of pain, also called persistent chronic pain memory (Apkarian et al. 2009). Experimental studies conducted on somatosensory plasticity of primate brain revealed that in light of limb amputation and peripheral nerve injury, neural reorganization in the subcortical region including brain stem and thalamus (ventroposterior nucleus, VP) acts as precursor, and plays an important role in the representational plasticity of somatosensory region (Jones 2000). Apart from that, recent studies on functional plasticity of primary somatosensory cortex (S1) demonstrate that this region memorizes the chronic pain quite efficiently (Brooks and Tracey 2005) and neuropathic pain like chronic-back-pain leads to decrement in prefrontal and thalamic grey matter density (Apkarian et al. 2004; Yi and Zhang 2011). Few other similar studies strongly suggest that thalamocortical processes have significant association with pathophysiology of pain. Other type of neuropathic pains such as orofacial pain also led to volumetric changes in grey matter of several brain regions such as S1, thalamus, anterior insular, putamen and nucleus accumbens. Those studies even went on to reveal that the mere stimulations from peripheral regions are not responsible for development and upkeep of chronic pain disorders, in fact functional and anatomical changes within higher brain regions might also be playing a prominent role in the process (Gustin et al. 2011). Hence, in this study the regions of S1 cortex and ventral posterolateral (VPL) nuclei of thalamus were chosen for putting probes for local field potential and spike train analysis, with the aim to reconstruct functional time-varying *mesoscale connectomes* from the brain activity in these regions. Each node in the network corresponds to a probe, and each link accounts for the functional association between the brain region activities registered in the surrounding of the respective probes. We deal with mesoscale connectomes because each probe samples the signal coming from a small cohort of neurons (in average from 3 to 5 neurons). Differently, micro-scale connectomes refer to networks where each node represents a probe that registers the activity of a single neuron.

## Methods

### Computational connectomics: Complex network analysis of brain connectivity

A connectome is an extensive map of neural connectivity of the brain. A connectome can be classified into two broad categories namely *structural* and *functional*. A structural connectome refers to the anatomic connectivity of the brain. It comprises of the white matter bulk, which establishes physical connectivity between different brain regions (Rubinov and Sporns 2010) representing a stable brain circuitry (Zippo and Castiglioni 2016). Techniques, which help in determining structural connectome of brain, are mostly tracing based methodologies e.g. diffusion imaging weighted techniques like diffusion tensor imaging (DTI). The functional connectome is the neural map of brain activity, which demonstrates a deviation from statistical independence between spatially separated brain regions. Some basic measures of this statistically dependent property are correlation, covariance, and spectral covariance and phase synchrony. It provides a dynamical representation of the brain activity and is essential in understanding the huge assemblage of transient behaviours (e.g. perception, cognition, reward etc.) (Zippo and Castiglioni 2016). Functional connectomes are constructed from data generated from functional neuroimaging techniques such as fMRI, EEG or MEG (Zippo and Castiglioni 2016). Structural and functional connectomes are represented by weighted symmetric adjacency matrix with each of the element of the matrix corresponding to a structural or functional feature between joining neurons or brain regions. These matrices can be truncated or binarized at certain thresholds based on the type of statistical attribute in consideration or the type of complex network analysis.

Extensive development and application of brain mapping techniques (few of them mentioned above) in recent years has generated massive amounts of brain connectome data that are both structural (anatomical tracts) and functional (spatiotemporal correlation in fluctuations) in nature. In order to make sense of such large amount of complex data from brain networks, several computational techniques have been developed in the past decade. The emerging research field that deals with these techniques is known as *brain connectomics* (Zippo and Castiglioni 2016). In brain connectomics the connectome is generally depicted as graph or network. However, here we propose our ‘sharp’ interpretation of the difference between the two terms. In mathematics, graph theory and theoretical informatics the term *graph* is generally preferred to emphasize the abstract origin. A graph does not need to emerge as a representation of the relations between parts of a concrete *dynamic* system, and it is a general and abstract representation of the connectivity relations between elements. In physics, engineering, social and economic sciences the term *network* is preferred. The term network is composed of two parts net-work and it refers to a graph that originates from the representation of the relations between *active* parts of a concrete system, which is characterized by *intrinsic dynamics*. Nevertheless, in this paper, in compliance with standard practice, the terms graph and network will be interchangeably used without any particular bias.

**Fig. 1:**
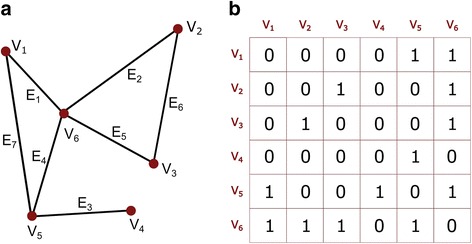
**a** An example of undirected and unweighted graph G = (V, E), where V is the set of nodes and E is the set of edges. **b** The binary adjacency matrix corresponding to the graph, where zeros and ones respectively represent the absence or presence of an edge between a pair of nodes

A graph *G* is a pair of sets (V, E). V is a set of vertices and E is a set of edges with each element of set E not connecting more than two elements of the set V. In Fig. 1a is shown a simple graph with V = {V1,V2,V3,V4,V5,V6} and E = {E1,E2,E3,E4,E5,E6,E7}.

The adjacency matrix (Fig. 1b) is a simple connection matrix representing what vertices are adjacent (or connected) to what other vertices (or nodes).The adjacency matrix of a graph G having N nodes is an N x N matrix. The rows and columns represent nodes of the graph and a particular matrix element (Vi,Vj) is marked as either 1 or 0 according to whether Vi and Vj are adjacent (or connected) or not. Figure 1 clearly illustrates interrelation between an unweighted graph and its binary adjacency matrix.

The graph in Fig. 1 can be interpreted as an example of a limited connectome. The nodes can be, for instance, the aggregated neuronal populations represented by the voxels (volume pixels) in the interconnected grey matter regions (V1,…,V6 according to the figure) and the edges can stand for either physical connections (binary values) or coherent activity levels between the nodes (fractional values). The body of knowledge, within which computational techniques to analyse and inspect complex networks are developed and applied, has emerged in the last decade and is known as complex network analysis (CNA). The fundamental aspects of CNA have been derived from graph theory, physics and engineering of complex systems. CNA of the neural connectivity stored in the connectome data (functional and structural) helps in identifying many significant features or proprieties, which are quantified in measures pertaining to the network topology and its global and local brain connectivity. Many of these topological measures are neuro-biologically meaningful and lead to the understanding of quantitative behaviour of the brain connectome (in our case, functional) in resting-state or under external stimuli. In this study, the entire story revolves around only one stimulus and that is neuropathic pain (Sporns 2011).

### Data acquisition and processing

One of the pain models of peripheral neuropathic pain involves chronic constriction injury (CCI) surgery (Bennett and Xie 1988; Jaggi et al. 2011; of the sciatic nerve in adult male Sprague-Dawly rat (Zippo et al. 2013). Local field potential (LFP) and spike train analysis (STA) data were acquired in particular days according to the procedure described in the paper authored by Zippo et al. (Zippo et al. 2013) for the times mentioned in Table 1 by invasive probing procedure into the two brain regions of one rat: primary somatosensory cortex (S1) and ventral posterolateral (VPL) nuclei of thalamus.

**Table 1:** Time steps of local field potential (LFP) and spike train analysis (STA) data recording from two brain regions (primary somatosensory cortex, S1, and ventral posterolateral nuclei of thalamus, VPL) of adult rat, after chronic constriction injury (CCI) surgery of the sciatic nerve

3 h post CCI	4 days post CCI	6 days post CCI	9 days post CCI	11 days post CCI	12 days post CCI	15 days post CCI	16 days post CCI

The LFP and STA data were obtained by 31 channels. Each channel sampled the signal provided by a probe inserted in S1 or VPL region that recorded the activity of either an individual or an aggregation of neurons (Zippo et al. 2013). Temporal coherence between LFP signals and STA data was detected by processing the recorded signals in overlapping time windows. These generated, for each time step in Table 1, a collection of 31 × 31 (31 network nodes: one for each probe) time-varying weighted (for functional association level) adjacency matrices. Specifically, for each time step, a signal of around 1 s was registered and this produced a total of 1000 short-time-varying adjacency matrices. The detailed procedure to pass from the 31 channel signals registered at a given time step, to the short-time-varying adjacency matrices is described in (Zippo et al. 2013). According to this procedure, out of 1000 adjacency matrices, only the middle 400 matrices have been taken into account for the analysis, since they are considered more reliable, whereas the first and last 300 have been discarded. These adjacency matrices represent the neural rewiring correlates of pain in the brain regions. Meanwhile, Von Frey test was performed at each time step on the subject rat for quantitative evaluation of the chronic neuropathic pain level. The Von Frey test measures as a mechanical threshold (maximal force) the behavioural response (withdrawal) to the stimulus of pain induced by a controlled mechanical stimulation: the poking of the rat’s hind paw by means of small pieces of nylon rod (Jaggi et al. 2011). The lower the value of the Von Frey test, the higher the pain sensitivity of the subject.

### Complex network analysis applied in our study

For each time step, for instance at 4 days post CCI, we obtain a collection of connectomes. The recording window is so narrow that we assume that ‘on average’ the collection of connectomes recorded inside that window represents a statistical sampling of the steady-state of the system at the considered time step. Since we can compute a measure that quantifies a particular topological feature of a connectome in a unique value, the average of these values - for the set of connectomes obtained at the considered time step - returns a *mean-description* of the system’s steady-state at that time step. This mean-description can be obtained for each time step in Table 1, resulting in a time-varying description of the mean connectome topological features. In practice, for each defined topological measure we can obtain a curve that progressively varies over the time steps, the ensemble of these curves represent different markers of topological features of the rewiring correlates of pain in the brain time-varying functional connectomes of the rat model after CCI. Since, a behavioural test (Von Frey test) was performed for each time step, a curve that offers a quantitative behavioural measure of the rat’s pain across the time steps is provided. To summarize, we have a total of 14 topological measure (whose description is offered in the section below) curves that quantify different respective features of the mean topological brain time-varying connectome re-wiring and 1 independent behavioural measure that quantifies the level of pain in the rat model.

The methodology of complex network analysis applied in this study is divided into two parts. The first part is the *correlation analysis* of each single topological network measure with the behavioural test. The second part is the *unsupervised comparative and comprehensive analysis* of the time-varying curves (topological and behavioural) all together.

### Correlation analysis of each topological network measure with the behavioural test

This first statistical analysis stems from the idea that if the topological measures are able to capture connectome’s topological features that are modified by the network rewiring correlates of pain, then the topological measure curves should correlate to a certain extent with the behavioural measure curve over time. In order to verify this hypothesis, we proceeded as follows.

1. Since the data points were sampled non-uniformly over time (3 h, 4 days, etc.), in order to obtain more refined and uniform steps we needed to reconstruct the signal at intermediate time points. In engineering and science it often occurs to have a discrete set of data points obtained by sampling. When the data are sufficiently accurate, the method used for estimating new points for intermediate values of the independent variable is called *interpolation* (Fritsch and Carlson 1980; Crochiere and Rabiner 1983).

In order to exclude any bias in the comparison between the topological measures and the behavioural test, the signals should be reconstructed in a totally independent way between each other, without making strong assumptions about the shape of the function, as nonparametric and data-driven algorithms do (Russell and Norvig 2009). An example of method that would not be suitable is linear *regression*, a parametric and hypothesis-driven approach for modelling the relationship between variables and for prediction tasks in supervised machine learning. In fact, it might force the topological measures and the behavioural test to fit the same trend, with the risk to introduce non-existent correlations given only by the fact that they fitted the same parametric model, potentially leading to false claims.

Furthermore, in order to avoid to construct information that is not present, we should rely as much as possible on the given data points. Therefore, we decided to adopt monotone piecewise cubic Hermite interpolation (PCHIP) (Fritsch and Carlson 1980), one of the most effective shape preserving interpolation, which is a data-driven, nonparametric and unsupervised procedure. Relying only on the accuracy of the data points, the worse is the reconstruction the worse will be the correlation between the interpolated signals, because a bad reconstruction can introduce only noise and it is known that the correlation between two noise signals is very low. For instance, the autocorrelation of a noise signal is almost zero for each time lag.

To conclude, this stringent choice - to prefer the use of interpolation (which is unsupervised, parameter-free and data-driven) to the use of a parametric regression (which is supervised, parameter-dependent and hypothesis-driven) - brings us in a safe zone in which either the signals are well reconstructed and the real correlations are obtained, or the signals are poorly reconstructed and any correlation potentially present is lost. In other words, it ensures that a high correlation between the reconstructed signals can only originate from a high correlation between the original signals and cannot be introduced by the interpolation procedure.

Additional file 1: Figure S1 highlights that the standard error of the topological measures for each time point (computed over 400 short-time-varying connectomes) is generally low enough to consider the uncertainty on the mean value negligible. Therefore, we can adopt the unique mean value as a reliable estimate to reconstruct the overall signal with an appropriate accuracy, in fact the main assumption for a correct usage of the interpolation procedure is the data reliability. The only exception is the standard error of the power-lawness for the time steps 4, 6 and 9 days. However, the error interval corresponding to the peak of acute pain (day 4) does not overlap with the following ones (days 6-9), therefore the main trend should not be affected.

The interpolation has been performed considering steps of one hour for the time range of the entire experiment. Therefore, the interpolated signal was a one-dimensional array with 382 values, one value for each hour from the 3rd hour of the 1st day to the 24th hour of the 16th day.

2. As second step, we computed both linear (Pearson) and nonlinear (spearman) correlations of each single topological measure curve versus the behavioural measure curve. This analysis explained the extent to which a certain topological measure is able to capture topological features whose variations in the brain networks are correlated with the presence of pain. In brief, it spots markers for the rewiring correlates of pain in the brain time-varying functional connectomes of a rat model. The following section offers details on the 14 considered topological measures.

### Statistical test for assessing the significance of the correlation between the interpolated signals

It might be argued that using the same interpolation method, to largely reconstruct from only 8 points a signal of even 382 points, could lead to an interpolation-bias, which could in theory deceptively increase the similarity between the reconstructed trends of a topological signal and the behavioural signal. In practice, this issue would generate an over-optimistic estimation of the correlations between the time trends of the topological signal and the behavioural signal. To express the same concept using simpler words, the risk might be that the high correlation is not due to the similarity between the real signals, but is rather due to the similar mathematical reconstruction (in our case PCHIP) adopted to fill large gaps between consecutive points in the two different signals. We stress that, according to the detailed explanation provided in the previous section, this argument is difficult to support in case of an unsupervised, nonparametric and shape preserving interpolation method such as the one adopted (PCHIP) to produce our results. However, we admit that it is crucial to control this degree of uncertainty. For such reason we offer below two different methodologies to assess and quantify how far from this risk are the values of correlation that we obtained using the interpolated signals.

To this aim, we invented a statistical test able to assess the significance of the correlation between the interpolated signals, testing the null hypothesis that a high correlation can be obtained by chance due to the interpolation procedure. If this is true, then an interpolated topological measure should likely obtain high correlation also with an interpolated *random* behavioural signal.

In order to test this, we invented two null-models for the behavioural signal. The first null-model consists in a Gaussian white noise, therefore the 8 values are random sampled from a standard normal distribution (Gaussian null-model, G). In the second null-model, instead, the 8 values are obtained by a random permutation (along the time direction) of the original Von Frey values (distribution-preserving null-model, DP). We created two null-models because, while the first model does not preserve the statistical distribution of the original signal, in the second null-model we wanted to be sure that also this constraint was indeed respected.

For a given topological measure the statistical test is performed as follows:

1. The 8 values of the topological measure and of the Von Frey test are interpolated and the correlation between the interpolated signals is computed.
2. The 8 values of the random behavioural signal are generated according to one of the two null-models (G or DP) and interpolated, then the correlation with the interpolated topological measure is computed. Repeating this step for a certain number of iterations *M* (we actually used *M* = 10,000), a null distribution of correlations is created.
3. A *p*-value for a two-tailed test is computed as:

$$ p={2}^{\ast}\min \left(\frac{\#\left[{C}_{rand}\le c\right]}{M},\frac{\#\left[{C}_{rand}\ge c\right]}{M}\right) $$

Where *c* is the observed correlation, *C*_*rand*_ is the set of *M* random correlations, and #[…] indicates the number of times in which the condition is verified.

The statistical test provides a *p*-value which indicates the probability to obtain a correlation of an interpolated topological signal with an interpolated *random*-behavioural-signal equal or more extreme than the one observed with the interpolated *real*-behavioural-signal. If the *p*-value is below the confidence level 0.05, we can reject the null hypothesis and accept the alternative hypothesis according to which the high correlation cannot be obtained by chance due to the interpolation procedure, therefore it is present in the real signals.

### Topological measures in complex and brain networks

The brain is anatomically and functionally segregated at multiple levels of organization and it consists of local collectives of strongly interconnected cells sharing inputs, outputs and response properties (Tononi et al. 1994). Specific anatomical areas for dedicated stimulus and response are also further segregated for more specific stimuli e.g. visual cortex responds to the visual stimulus in functionally segregated manner for different aspects of vision such as colour, motion, form etc. From the perspective of CNA, functional brain segregation can relate to the local-derived concept of average clustering coefficient of the functional connectome.

Despite of such spatial segregation, the brain demonstrates global functional integration in various aspects, for instance combining specialized information from different regions to provide a unitary behavioural output, which reflects a coherent response to the integration and combination of multiple local processes. Functional integration can relate to the global-derived concept of average path length in the functional connectome. Specifically, in the structural connectomes, path length means combination of nodes and links resulting in physical information flow; whereas in the functional connectomes, path length means a sequentially coherent statistical relation between subsequent regions, and might not be always supported by physical information flow through anatomical connections.

In complex network analysis, a topological network measure quantifies by means of a unique numerical value the extent to which a certain mechanism of organization (or topological feature) influence the network connectivity. For instance, many network measures take into account mechanisms of segregation or integration of the different parts of a complex network. Network measures can be *stochastic* or *deterministic*. Stochastic measures involve the generation of randomized networks (a null model of a given network) based on some topological characteristics preserved from the original networks. These randomized networks are used to evaluate the prevalence that a certain topological mechanism of organization shows in the original network in respect to the randomized model of the same network. These randomized (but characteristically preserved or bounded) networks induce the stochasticity in the output of the measure that occurs because the inherent creation of these null models is stochastic. The stochastic measures included in the present study are five: small-worldness (two different types), power lawness, modularity and structural consistency. Deterministic measures are based on the direct quantification of a considered network topology feature (or rule of organization) e.g. node degree. Some of these measures are even calculated on the basis of other deterministic measures. The randomized networks, which represents the null models, are not required to evaluate deterministic measures: hence, the numerical value associated with the measure evaluated for a given network is always the same. This often implies that the *computational time* of deterministic measures is in general shorter than the one required by stochastic measures. The deterministic measures included in the present study are nine: average node degree, characteristic path length, average clustering coefficient, efficiency, closeness centrality, node betweenness centrality, edge betweenness centrality, radiality and local-community-paradigm correlation. A detailed description of both stochastic and determinist measures is provided below.

In addition, a topological measure can be either *local* or *global*. It is local if it makes a statistic evaluation of local topological information in the neighbourhood of a node or a link. It is global if it makes a statistical evaluation of global topological information that emerges from nodes or links that are not in a neighbourhood. Note that for neighbourhood we intend the ensemble of nodes that are first-neighbours of a given node or edge. We will specify whether a measure is local or global in detailed description below.

### Stochastic measures

Since the stochastic measures provide different results if run multiple times, in order to control the stability of their output we executed a stability check. This check was performed to evaluate the convergence of stochasticity of these measures and determine their settling values. For this purpose, each measure was evaluated multiple times and a mean value was computed for all the iterations along with standard error. We found that the standard error was very small in comparison to the mean value of each measure, and for this reason we concluded that all the measures were converging to a correct mean value. Here below we discuss the five stochastic measures included in the present study: small-worldness (two different types: sigma ‘σ’ and omega ‘ω’), power lawness, modularity and structural consistency.

The *small-wordness (SWσ and SWω)* (Humphries and Gurney 2008; Telesford et al. 2011) integrates a local and global measure and was proposed for the characterization of a given network as small world. It relies on comparing a given network with an equivalent random network and lattice network on the basis of average clustering coefficient and characteristic path length. In 2006 a measure called ‘σ’ for characterizing small world networks was introduced by Humphries et al. (Humphries and Gurney 2008). To calculate this measure, the average clustering coefficient C and characteristic path length L of the network are measured and compared with respect to C and L of an equivalent random network (with similar node degree distribution) and the ratio is determined as follows. *γ* = *C*/*C*_*rand*_ and *λ* = *L*/*L*_*rand*_. These ratios give the small world coefficient *σ* = *γ*/*λ*. A condition for a network to exhibit small world features is that the characteristic path length should be close to that of an equivalent random network, *L* ≈ *L*_*rand*_. And the average clustering coefficient should be close to that of an equivalent lattice network, which also implies that C should be much higher than that of equivalent random network, *C* ≫ *C*_*rand*_. These boundary conditions, if met, restrict the value of *σ* > 1 for small world networks. The problem with this measure is that even small variations in the already low value of the average clustering coefficient for random networks, *C*_*rand*_, significantly influences the value of *γ*. To overcome this problem, a new robust measure was introduced by Telesford et al. (Telesford et al. 2011) which is called ‘*ω*’. Since *L* of a small world network is near to *L*_*rand*_ of an equivalent random network, and *C* of a small world network is near to *C*_*latt*_ of an equivalent lattice network, in ‘*ω*’ the ratio of the average clustering coefficient is calculated with respect to an equivalent lattice network and the ratio of the characteristic path length is calculated with respect to an equivalent random network:

$$ \omega =\frac{L_{rand}}{L}-\frac{C}{C_{latt}} $$

Hence this measure neglects fluctuations of *C*_*rand*_. Since for small world networks boundary condition is that *L* ≈ *L*_*rand*_ and *C* ≈ *C*_*latt*_, the values of *ω* come out to be near 0 for small world networks. The equation suggests that *ω* ∈ [−1, 1], with positive values suggesting the network having more randomness, *L* ≈ *L*_*rand*_ ∧ *C* ≪ *C*_*latt*_, and negative values suggesting network to be more latticed, *L* ≫ *L*_*rand*_ ∧ *C* ≈ *C*_*latt*_. Brain functional connectomes are generally small-world.

The *power-lawness (PL)* (Clauset et al. 2009) is a local measure that offers a confidence level to claim whether power-law node distribution governs a particular network or not. If a network follows this law, it can be considered as scale free network. To ensure power law as a plausible hypothesis, *p*-value is calculated according to the method described in Clauset et al. (Clauset et al. 2009). If the calculated *p-*value ≥0.1, the power law hypothesis is accepted for the network, otherwise rejected. In normal conditions, brain connectomes do not show scale-free distribution (Gastner and Ódor 2016)

The *modularity* (*Q)* is a global measure, and quantifies (Newman 2004; Newman 2006) the tendency of a network to be decomposed into segregated modules or communities. In networks with high modularity, the modules tend to interact densely within themselves but interact sparsely or not at all with the other modules. Modularity index *Q* measures the goodness of the best possible partition of nodes, which maximizes the intra-modular connectivity and minimizes the inter-module connectivity. It is computed by the following formula.

$$ Q=\sum_{u\epsilon M}\left[{e}_{u u}-\kern0.5em {\left(\sum_{v\epsilon M}{e}_{u v}\right)}^2\right] $$

Where *M* is the partition of the nodes (whose elements *u* are called modules) and *e*_*uv*_ is the proportion of links in module *u* connecting to module *v*. Modularity can assume either a positive or a negative value, and lies in the range [−1/2, 1). Positive modularity suggests presence of distinct community structure in the network, and values greater than about 0.3 appear to indicate significant community structure (Newman 2004). Most of the times, average clustering coefficient and modularity show correlation with each other.

The *structural consistency (SC)* (Lü et al. 2015) is a global measure and a quantitative index for measuring the link predictability of a complex network. The link predictability quantifies the inherent facility to predict the missing or non-observed links of a complex network regardless of the specific algorithm used for the prediction. SC relies on the random perturbation (which is origin of stochasticity) and first-order approximation of the adjacency matrix. The hypothesis on which is based this measure suggests that a group of links is predictable if removing them has only a small effect on the network structural features. In fact, the topological regularity of a network is reflected in the consistency of structural features before and after a random removal of a small set of links (Lü et al. 2015). This measure exists in the interval [0,1], where 0 indicates absence of link predictability and 1 indicates full link predictability.

### Deterministic measures

The *average node degree (AND)* is a local measure and the simplest indicator of node centrality in the network, it is defined as:

$$ AND=\sum_i\ \frac{d_i}{n} $$

Where N is the set of all the network nodes, d_i_ is the node degree of each node *i* ϵ N and *n* is the size of the set N.

The *average shortest path distance a.k.a. characteristic path length (CPL)* (Watts and Strogatz 1998; Telesford et al. 2011) is a global measure and describes the average of all shortest path lengths between all the pairs of vertices. It can be calculated by summing all the shortest path lengths between pairs of nodes divided by the total number of pairs of nodes *{i,j}.*

$$ CPL=\sum_{i<j}\frac{sp_{i,j}}{n\bullet \left(n-1\right)/2} $$

Where *sp*_*ij*_ is the shortest path length between a unique pair of nodes *i, j* ϵ N. A small value of characteristic path length of a connectome means that the information flow between the nodes across the network is facilitated, and that the nodes are able to send messages with each other easily. In other words the nodes across connectomes are functionally convergent.

The *average efficiency (AE)* (Latora and Marchiori 2001; van den Heuvel et al. 2009) is a global measure and quantifies how efficiently the information is exchanged within the network. It is inversely proportional to the CPL. If the CPL is low then efficiency is high.

$$ AE=\sum_{i<j}\frac{2}{n\bullet \left(n-1\right)\bullet {sp}_{i,j}} $$

The *average clustering coefficient (ACC)* (Watts and Strogatz 1998) is a local measure and offers an average evaluation of the cross-interaction density between the first neighbours of each node in the network.

$$ ACC=\frac{1}{n}\sum_i\frac{t_i}{k_i\bullet \left({k}_i-1\right)/2} $$

Where *t*_*i*_ is the number of cross-interactions that occur between the first neighbours of the node *i* ϵ N. The denominator is the total number of possible cross-interactions that could occur between the first neighbours of the node *i* ϵ N*.* Large values of this measure indicate that the nodes in the network tend to have highly connected neighbours.

The *average closeness centrality (ACCE)* (Bavelas 1950; Sabidussi 1966) is a global measure, represents an indicator of node centrality, and calculates the average closeness of the nodes from all the others in the network. The closer a node is to the others, the faster it can spread information to the others sequentially. It is calculated by averaging all the reciprocals of the mean shortest distance of a particular node to all other nodes.

$$ ACCE=\frac{1}{n}\sum_i\frac{n-1}{\sum_{j\ne i}{sp}_{ij}} $$

In brain connectomics, if the average closeness centrality of the network is low then the activity of each node would be functionally more relevant to the other nodes.

The *average node betweeness centrality (ANBC)* (Brandes 2001) is a global measure and also a node centrality indicator. The single node betweeness centrality measures how crucial is a particular node in maintaining an optimum information flow path between any other node pair. In other words this average measure calculates the average stress of information burden on the network nodes. It is defined as:

$$ ANBC=\frac{1}{n}\sum_{j\ne i\ne k}\frac{\sigma {(i)}_{jk}}{\sigma_{jk}} $$

Here: *i, j,k* ϵ N and σ(i)_jk_ is the number of shortest paths between *j* and *k* that pass through *i*; σ_jk_ is the total number of shortest paths between *j* and *k.*

The *average edge betweeness centrality (AEBC)* (Brandes 2001) is a global measure and an edge centrality indicator. The single edge betweenness centrality measures the betweenness or information stress on an edge. The average measure calculates the average stress of information burden on the network edges. The mathematical formula is identically to the ANBC but it should be adjusted considering the edges (and their total number) instead of the nodes.

The *average radiality (AR)* (Isik et al. 2015) is a global measure and an indicator of centrality. It expresses the average tendency of proximity (or isolation) of a node to the other network nodes. For a single node, it measures the ease of that node to be reached by other nodes.

$$ AR=\frac{1}{n}\sum_i\frac{\sum_{j\ne i}\left(D+1-{s}_{ij}\right)}{D-1} $$

Where D is the diameter of the network and indicates the length of the maximum network shortest path.

**Fig. 2:**
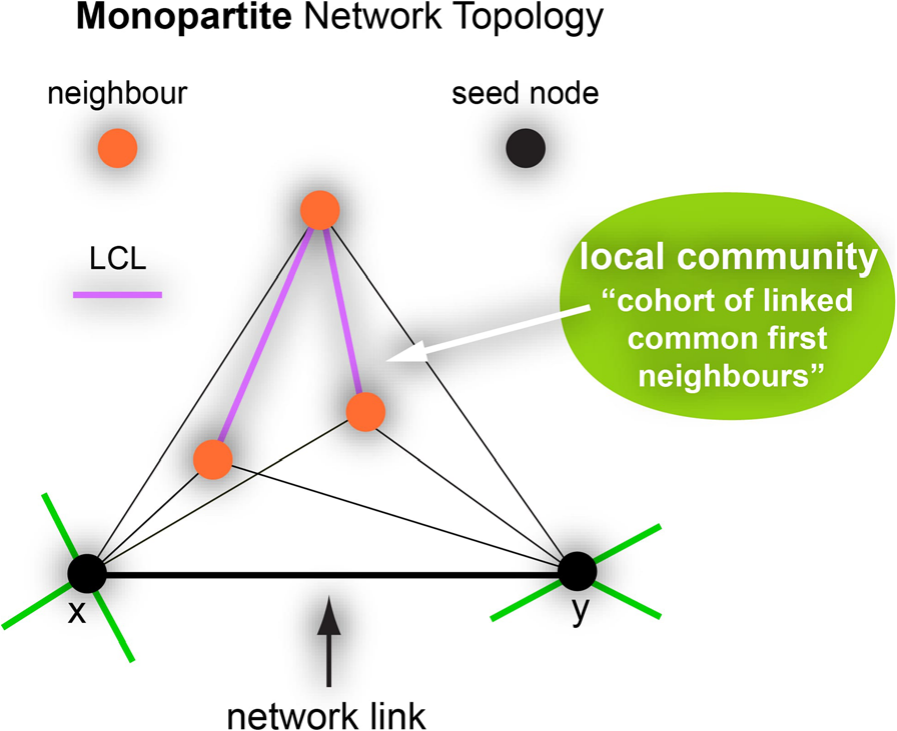
The figure is a visual representation of the LCP-theory proposed to model local-topology-dependent link-growth in complex networks. Let’s focus on a network link between two seed nodes X and Y (*black*). Nodes that are first-level neighbours of the seed nodes and that are shared between them represent their common neighbours (*CN, orange*). In green are shown links to first-level neighbours that are not shared. The cohort of the CNs together with their cross-interactions, named local community links (*LCL, purple*), form a local community. According to the *LCP-theory*, in many complex networks there is a high positive correlation between the number of CNs and the number of the corresponding LCLs for each link in the network, and both the information should be exploited in order to explain the local-topology-based emergence of links in the network that is named *epitopological learning*

In contrast to the existing node-neighbourhood-based local measures, a new strategic shift has been introduced recently in which the focus is no longer only on groups of common nodes and their node neighbours, but also on the organization of the links between them (Cannistraci et al. 2013a). This new idea inspired a theory, which is known as the local community paradigm (LCP-theory) and is valid both in monopartite (Cannistraci et al. 2013a) and in bipartite (Daminelli et al. 2015; Durán et al. 2017) undirected unweighted networks. The *LCP-theory* was proposed to mechanistically and deterministically model local-topology-dependent link-growth in complex networks, and holds that for modelling link prediction in complex networks, the information content related with the common neighbour nodes (CNs) of a given link should be complemented with the topological information emerging from the interactions between them (Fig. 2). The cohort of CNs and their cross-interactions – which are called local community links (LCLs) - form what is called a local community (Fig. 2). This first part of the theory inspired the Cannistraci variation of the classical CN-based similarity indices for link prediction, named also LCP-based link predictors. Since this subject is not in the scope of this article, for details refers to Cannistraci et al. (Cannistraci et al. 2013a; Daminelli et al. 2015; Durán et al. 2017).

Furthermore, the LCP-theory holds that in many complex network topologies, the number of CNs of each link in the network is positively correlated with the respective number of LCLs. This second part of the LCP-theory motivated a new network measure called *local-community-paradigm correlation (LCP-corr)* (Cannistraci et al. 2013a; Daminelli et al. 2015; Durán et al. 2017), which is a local measure that represents an exception in respect to the majority of the previous ones, for two main reasons. Firstly, it is not related with only the node neighbourhood but with the node/link neighbourhood. Secondly, the general statistic used to obtain a unique value is not the average but the Pearson correlation. The formula for computing the LCP-corr is:

$$ LCPcorr=\frac{\mathit{\operatorname{cov}}\left( CN, LCL\right)}{\sigma_{CN}.{\sigma}_{LCL}}, when\  CN>1 $$

where *cov* indicates the covariance operator and σ the standard deviation. This formula is clearly a Pearson correlation between the CN and LCL variables. CN indicates a one-dimensional array. Its length is equal to the number of links in the network that have more than one common neighbour, and it reports the number of common neighbours for each of them. LCL indicates a one-dimensional array of the same size of CN, and it reports the number of local community links between the common neighbours. Mathematically the value of LCP-corr would be in the interval [−1,1]. But, extensive tests on many artificial and real complex networks demonstrate that an inverse correlation between CN and LCL is unlikely, therefore the interval is in general between [0,1]. In particular, it was revealed that LCP networks (with high LCP-corr, i.e. > 0.7) are very frequent to occur, and they are related to dynamic and heterogeneous systems that are characterised by weak interactions (relatively expensive or relatively strong) that in turn facilitate network evolution and remodelling. These are typical features of social and biological systems, where the LCP architecture facilitates not only the rapid delivery of information across the various network modules, but also the local processing. In contrast, non-LCP networks (with low LCP-corr, i.e. < 0.4) are less frequent to occur and characterise steady and homogeneous systems that are assembled through strong (often quite expensive) interactions, difficult to erase. This non-LCP architecture is more useful for processes where: i) the storage of information (or energy) is at least as important as its delivery; ii) the cost of creating new interactions is excessive; iii) the creation of a redundant and densely connected system is strategically inadvisable. An emblematic example is the road networks, for which the money and time costs of creating additional roads are very high, and in which a community of strongly connected and crowded links resembles an impractical labyrinth.

In normal conditions, brain connectomes follow LCP organization (Cannistraci et al. 2013a), therefore they are characterized by high LCP-corr, which is in general higher than 0.8.

### Unsupervised comparative and comprehensive analysis of the time-varying curves

This second statistical analysis is unsupervised, therefore data driven. Differently from the first statistical analysis, it does not stem from any particular hypothesis that is a-priori defined. The fact that the sample labels (if known) are not used for the data projection make the analysis unsupervised.

The common practice in unsupervised dimension reduction analysis of datasets is to consider only the first two (or three, less used) dimensions of mapping, and the goal is to visually explore the distribution of the samples and the incidence of significant patterns. This procedure is useful to obtain unbiased (as we said the labels are not used in the algorithm learning phase) confirmation of the separation between groups of samples for which a possible diversity is theorized or expected. Specifically, in our study, the aim is to visualize and investigate the intrinsic relation of similarity that unsupervisedly emerge between the 15 time-varying signals: 14 network-based (topological measures at each time step) and 1 behavioural-based (Von Frey test at each time step). Therefore, in the two-dimensional reduced space of representation, signals similar to the Von Frey test should be mapped in a position close to the Von Frey test. And, in general, signals with similar trend should be mapped close to each other.

We created a dataset where the samples are the 15 time-varying interpolated signals and the features are the 382 values that these signals assumed at the different hour time steps. Each of the time-varying signals was z-scored (subtraction of the mean followed by division for the standard deviation of the signal) in order to scale them in the same range. The signals having inverse (negative) correlation in comparison to the Von Frey test were inverted by multiplication for −1, in order to adjust the mismatching due to the anti-correlation.

We performed a linear and nonlinear multivariate analysis (dimensionality reduction) by means of two parameter-free unsupervised machine learning algorithms: principal components analysis (PCA) for the linear analysis and Minimum Curvilinear Embedding (MCE) for the nonlinear analysis. We chose PCA because it is the mainstream multivariate method to unsupervisedly explore data patterns in multidimensional data (Ringner 2008). For comparison, we chose MCE (Cannistraci et al. 2010; Cannistraci et al. 2013b), a nonlinear version of PCA that is still parameter-free (an important advantage in unsupervised analysis) and that demonstrated to achieve top performance in unfolding patterns in many applications from biology and medicine to radar signal analysis. Therefore, MCE was considered an ideal version of a nonlinear-PCA to apply in this complementary analysis. In PCA, the dimensions emerge as the unsupervised inference of a new system of coordinates, which represent orthogonal directions that maximize and compress uncorrelated sample variance in the multidimensional data. For clarity, each dimension is a new meta-feature that is the linear combination of the original features. These dimensions are ranked in order of decreased variance, and the first two dimensions account for the highest variance in the multidimensional space. The patterns (where they exist) that naturally emerge in the first two PCA dimensions represent and visualize intra-similarity and inter-variability between groups of samples. Therefore, the sample separation is related with higher linear similarity and variability present in the data. MCE, on the other hand, is a type of nonlinear and parameter-free PCA that performs unsupervised inference of the directions that maximizes orthogonal patterns of nonlinear and hierarchical organized data variability/similarity. Using the MCE algorithm, we searched for the hidden pattern (specifically: the ordering of the signals on one of the first two dimensions) that explains the higher nonlinear variability in the data, and next we compared this ordination with the signal labels (for instance, the positions of the topological measures in respect to the Von Frey test). More specifically, the dataset was analysed using non-centered Minimum Curvilinear Embedding (MCE), which is a form of parameter-free nonlinear-kernel principal component analysis (PCA) designed for nonlinear dimensionality reduction (Cannistraci et al. 2010; Cannistraci et al. 2013b). The principle behind MCE, minimum curvilinearity, suggests that curvilinear distances between samples (here, the experimental conditions) may be estimated as pairwise distances over their minimum spanning tree constructed according to a selected norm (in our case, Euclidean distance) in a high-dimensional feature space (here, the values of the signals at the different time steps). The collection of all nonlinear pairwise distances forms a distance matrix (the MC-kernel), which is next factorized by singular value decomposition and embedded in a two-dimensional space for visualization and analysis (Cannistraci et al. 2010; Cannistraci et al. 2013b). MCE required a quantile normalization of samples in the dataset, in order to approximate the sample values to a similar distribution.

## Results and discussion

**Fig. 3:**
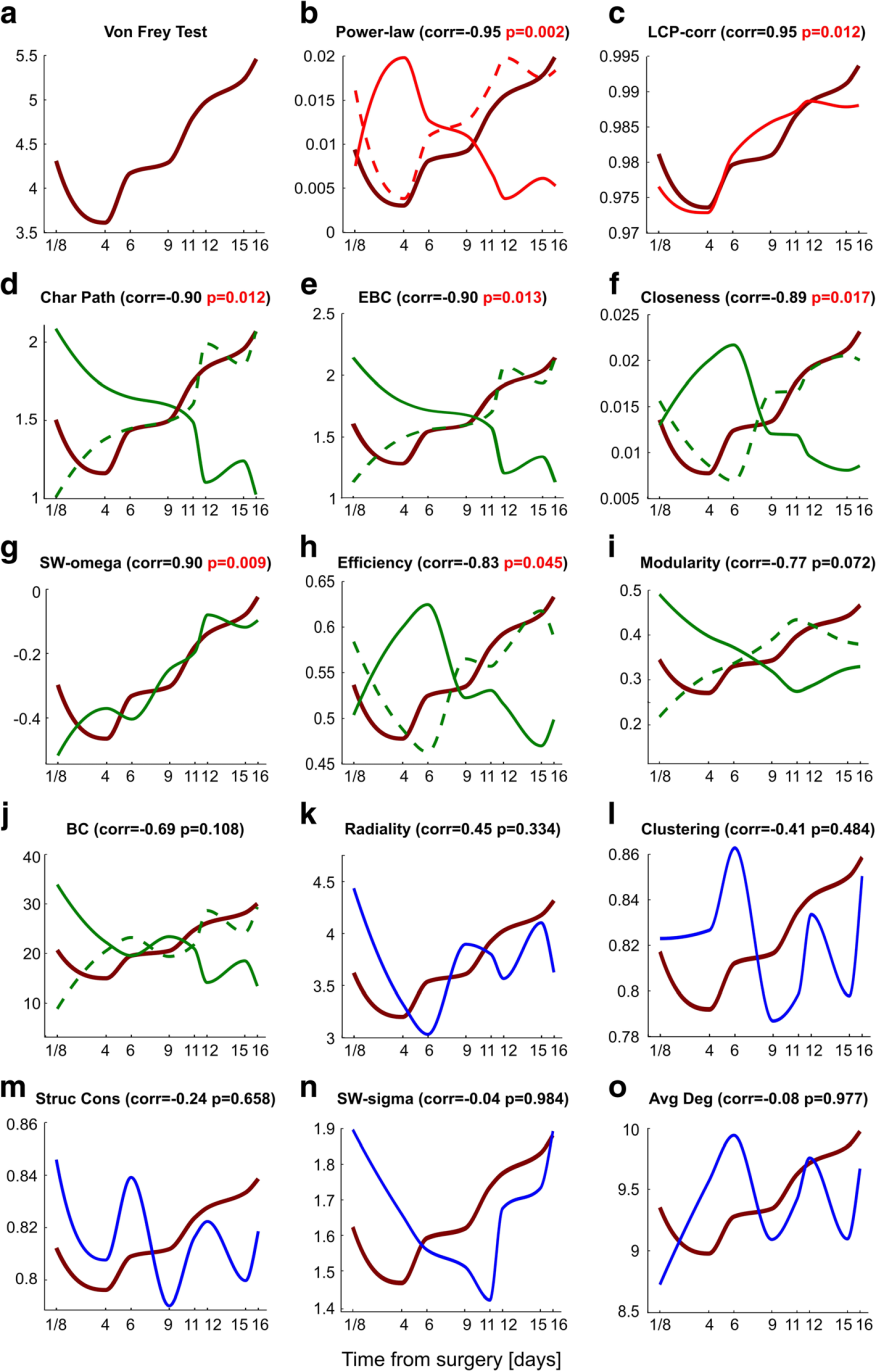
The figure shows the evolution in time for a measure of the Von Frey behavioral test in comparison to topological measures of the connectome, during consecutive time steps after CCI surgery in which LFP data were recorded. In all the plots the x-axis represents the time elapsed after the surgery and the distance between adjacent ticks is proportional to the actual time between those time steps. **a** The plot shows the Von Frey test, a behavioral test for measuring mechanical allodynia (at a certain time). The y-axis indicates the measure of maximal force (in grams) before the withdrawal of ipsilateral paw. **b**-**o** The plots highlight the comparison between the Von Frey test (shown in *brick red*) and every topological measure. The correlation between each topological measure with respect to the Von Frey test is reported, together with the *p*-value of the statistical test for assessing the significance of the correlation. Note that the values correspond to the columns “Max correlation” and “Max *p*-value” of Table 2. Significant *p*-values are highlighted in red. The topological measures (**b**) Power Lawness and (**c**) LCP-correlation demonstrate high correlations (shown in *red*), 0.9 < corr ≤1. The measures (**d**) Characteristic Path Length, (**e**) Edge Betweenness Centrality, (**f**) Closeness Centrality, (**g**) Small Worldness ω, (**h**) Efficiency, (**i**) Modularity and (**j**) Betweenness Centrality demonstrate medium correlation (shown in green), 0.5 < corr ≤0.9. The measures (**k**) Radiality, (**l**) Clustering Coefficient, (**m**) Structural Consistency, (**n**) Small Worldness σ and (**o**) Average Degree are least correlated (shown in *black*), 0 ≤ corr ≤0.5. The Von Frey values have been rescaled before being superimposed in every plot in order to visually highlight the correlation. When the correlation is negative and medium-high in absolute value, the topological measure inverted plot is also shown with a dotted line. Additional file 1: Figure S1 reports the error intervals for the mean values of the topological measures at each time step, highlighting that the data is reliable enough for a correct usage of the interpolation procedure

The first important discovery of this study is that half (7 on 14) of the total number of topological measures, considered for characterizing the time-varying rewiring of the network, showed a high (≥0.8) correlation (either Pearson or Spearman) with the behavioural test for pain (Fig. 3, Table 2). Furthermore, these 7 measures obtained a significant *p*-value (≤0.05) in the statistical test for assessing the significance of the correlation. As a consequence we can reject the null hypothesis and accept the alternative hypothesis according to which the high correlations cannot be obtained by chance due to the interpolation procedure, therefore these correlations seem to derive from the trends of the real signals. We notice that the significance of these high correlations is confirmed using two different null-models (Table 2), which are the Gaussian white noise (G) and the distribution-preserving (DP). Examples of statistical tests for the two measures that obtained the highest correlations, power-lawness and LCP-corr, are shown in Additional file 1: Figure S3. Finally, although the PCHIP is the most appropriate interpolation algorithm, as already discussed in the Methods section, we have repeated the same analysis using different interpolation techniques to reconstruct the behavioural signal (both the real signal of the experiments and the random signal of the null-models). Additional file 1: Tables S1-S2 highlight that if either spline or linear interpolation are adopted, the results are very similar to the ones shown in Table 2, where PCHIP is used. This test is an additional proof to conclude that, not only the high correlations cannot be generated by chance due to the interpolation procedure, but they are also robust with respect to the adoption of different algorithms to interpolate the behavioural signal.

**Table 2:** The table provides the results for the correlation analysis of the topological network measures with the behavioural test and for the related statistical test

	Max correlation	Max *p*-value	Pearson correlation	Spearman correlation	Pearson *p*-value (G)	Spearman *p*-value (G)	Pearson *p*-value (DP)	Spearman *p*-value (DP)
Power-law	−0.954	**0.002**	−0.954	−0.941	0.001	0.003	0.002	0.004
LCP-corr	0.949	**0.012**	0.908	0.949	0.012	0.012	0.010	0.010
SW-omega	0.899	**0.009**	0.899	0.823	0.007	0.007	0.009	0.005
Char Path	−0.898	**0.012**	−0.887	−0.898	0.009	0.012	0.012	0.009
EBC	−0.898	**0.013**	−0.884	−0.898	0.009	0.013	0.012	0.011
Closeness	−0.891	**0.017**	−0.872	−0.891	0.013	0.017	0.010	0.013
Efficiency	−0.827	**0.045**	−0.793	−0.827	0.042	0.045	0.046	0.041
Modularity	−0.774	0.072	−0.743	−0.774	0.067	0.070	0.080	0.072
BC	−0.690	0.108	−0.689	−0.690	0.083	0.108	0.077	0.105
Radiality	0.446	0.334	0.391	0.446	0.344	0.330	0.349	0.334
Clustering	−0.410	0.484	−0.305	−0.410	0.474	0.469	0.481	0.484
Struc Cons	−0.238	0.658	−0.169	−0.238	0.661	0.658	0.644	0.642
Avg Deg	−0.083	0.977	−0.002	−0.083	0.983	0.971	0.964	0.977
SW-sigma	−0.041	0.984	0.012	−0.041	0.988	0.978	0.981	0.984

For each topological measure, both the Pearson and the Spearman correlation computed with the Von Frey test are reported. For each of the two correlations, the *p*-value of the statistical test is shown for highlighting the significance of the correlation. The *p*-values suffixes G and DP refer respectively to the first (Gaussian white noise) and second (distribution-preserving) null-models. The first two columns are intended to summarize the results: “Max correlation” reports the best among the two correlations between Pearson and Spearman, whereas “Max *p*-value” indicates, for that correlation, the maximum between the two *p*-values obtained with the two null-models (in order to be more conservative). The significant *p*-values in this column, considering a confidence level of 0.05, are highlighted in bold

The fact that 7 measures were able to detect a significant variation associated to pain confirms and preciously extends the results of previous studies (Zippo and Castiglioni 2016), showing that the presence of pain triggers major changes in the rewiring of the functional S1-VPL network. However, 5 of these 7 measures achieved a correlation with the behavioural test for pain that was ≥0.8 and ≤0.9, and interestingly they were all global connectivity topological measures. The *characteristic path length* represents the most relevant of them since it achieved Spearman correlation 0.9. It tends to increase almost monotonically from 3 h to 16 days after surgery. This suggests that in the network the average shortest path separation between the nodes increased regardless of the acute or chronic phase of the pain. However, none of the 5 global measures in the 0.8 to 0.9 interval of correlation showed a distinctive ability to detect and to be in phase with the evident peak of acute pain that is revealed by a significant reduction of the behavioural test value at 4 days after surgery. This suggests that acute pain might affect the global network connectivity only marginally.

**Fig. 4:**
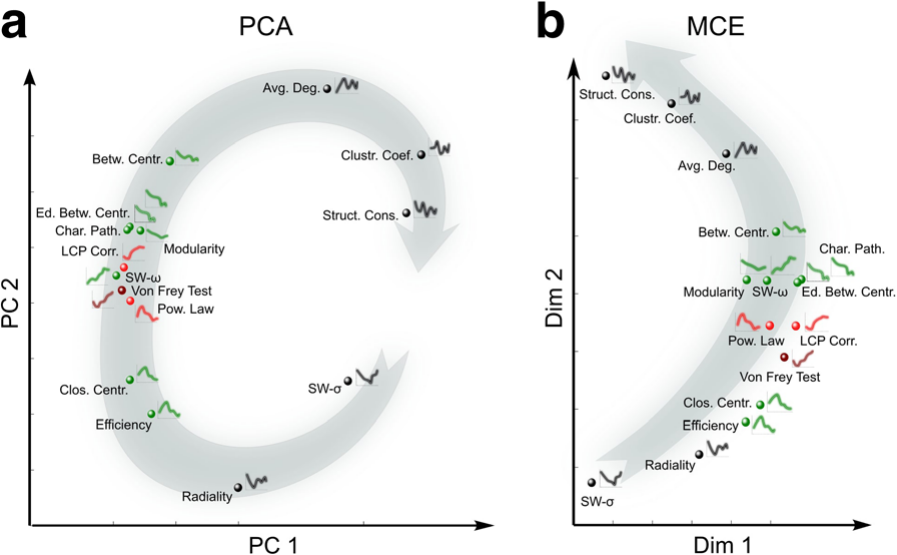
The figure shows two methods for linear and nonlinear multivariate analysis: (**a**) Principal Component Analysis (PCA) and (**b**) Minimum Curvilinearity Embedding (MCE). PCA is a method for linear dimensionality reduction and the points on the PCA plot (representing the various topological measures) are distributed according to a circular pattern, which suggests the presence of non-linear relationships among the data points. MCE is a non-linear method for dimensionality reduction, therefore the data points are distributed more linearly compared to PCA. The X and Y axes of (**a**) are the principal components (PC) 1 and 2 respectively. The X and Y axes of (**b**) are the dimensions 1 and 2 of the embedding. Since multivariate analysis considers the overall strength of relationships between each pair of variables (individual data points of each plot), the topological measures with curves of similar shape tend to be closer in the reduced space. For instance, LCP-correlation and Power Lawness, which have high correlation values and a similar shape with respect to Von Frey test, are also close to each other. Points corresponding to topological measures with medium-high correlation values are shown in the same color as in Fig. 3, whereas measures in the low correlation range are colored in *black*

Surprisingly, the second important finding of this study is that the only two measures to provide a very high correlation (>0.9) with the behavioural test were local measures: power-lawness and LCP-corr (Fig. 3). This indicates that the local network features detected by these two different topological measures were associated with rewiring during both the acute and the chronic pain phase. The same results were confirmed also using the *unsupervised comparative and comprehensive analysis* of the time-varying curves (topological and behavioural) by means of the PCA and MCE dimension reduction (Fig. 4). PCA (Fig. 4a) is a linear transformation and cannot solve the nonlinear similarity relations between the signals, in fact they appear circularly (nonlinear shape) ordered according to their similarities. Nevertheless, the profit of this analysis is that, although the nonlinear similarity relations were not linearized on one unique dimension of embedding, and two dimensions were necessary to obtain the signals’ segregation, the accuracy of this segregation is in manifest accordance with the result of the previous correlation analysis. Apart from a minor error of mapping for the SWω, all the other signals are properly mapped, suggesting that also an unsupervised analysis can confirm the finding we obtained. This is even more crystal-clear in the mapping provided by MCE (Fig. 4b), which is more accurate than PCA because MCE is a nonlinear machine learning for dimension reduction. The signals (Fig 4b) are correctly and almost linearly ordered according to a relation of progressive similarity that is coincident with the second dimension of embedding. Interestingly, the average clustering coefficient attained low correlation values with the behavioural test (it is indeed located far from the Von Frey test also in the dimension reduction mappings in Fig. 4) indicating that looking at the average connectivity between the neighbours of a node should not be a preferred strategy to reveal local dynamical network changes related with pain. On the other hand, looking at the connectivity between the neighbours of a link (Fig. 2) seems a proper strategy to reveal the local network remodelling associated with pain, in fact the LCP-corr is a topological measure designed to capture this type of local information in the network topology. The high correlation obtained by the LCP-corr can be explained by the *epitopological learning*, which is the intuition that inspired the foundation of the LCP-theory (Cannistraci et al. 2013a).

In 1949, Donald Olding Hebb advanced a *local learning rule* in neuronal networks that can be summarized in the following: neurons that fire together wire together (Hebb 1949). In practice, the Hebbian learning theory assumes that different engrams (memory traces) are memorized by the differing neurons’ cohorts that are co-activated within a given network. Yet, the concept of wiring together was not further specified and could be interpreted in two different ways. The first interpretation is that the connectivity already present - between neurons that fire together - is reinforced; whereas, the second interpretation is the emergence and formation of new connectivity between non-interacting neurons already embedded in an interacting cohort.

The first interpretation has been demonstrated in several neuroscientific studies, where it was proven that certain forms of learning consist of synaptic modifications, while the number of neurons remains basically unaltered (Ansermet and Magistretti 2007; Corti et al. 2008; Ziv and Ahissar 2009). A first mathematical model of this learning process was implemented in the Hopfield’s model of associative memory, where neuron-assemblies are shaped during engram formation by a re-tuning of the strengths of all the adjacent connections in the network (Baldi and Sadowski 2016). It is important to specify that neuronal networks are over-simplified models and between two nodes (that represent two neurons) only one unique connection, which is deceptively called ‘synapsis’, is allowed. This unique artificial synapsis is a network link with a weight (or strength) and abstractly represents in a unique connectivity all the multitude of synapses that can occur between two real neurons in a brain tissue. For non-computational readers, we stress that the word ‘synapsis’ used in computational modelling of artificial neural networks might be misleading for neurobiologists, and should be intended as a mere link between two nodes of a network that comprehensively symbolizes the strength of all the real biological synapses connecting two neurons. Here, and in the remainder of this article, we will refer only to this artificial neural network model where a link between two nodes (neurons) indicates an abstract interaction between them. In fact, although this artificial network model is based on evident simplifications, it demonstrated to be a powerful tool to simulate learning processes of intelligent systems (Baldi and Sadowski 2016; Baldassi et al. 2016).

Surprisingly, the second possible interpretation of the Hebbian learning - a cohort of interacting neurons that fire together give rise to new connections between non-interacting neurons in the cohort - to the best of our knowledge was never formalized as a general paradigm of learning, and therefore, it was never used with success to modify the architecture of abstract neural networks to simulate *pure topological learning*. We acknowledge the existence of studies that investigate how neuronal morphology predicts connectivity (Rees et al. 2017). For instance, Peters’ rule predicts connectivity among neuron types based on the anatomical colocation of their axonal and dendritic arbors, providing a statistical summary of neural circuitry at mesoscopic resolution (Rees et al. 2017). However, no paradigms were proposed to explain the extent to which new connections between non-interacting neurons could be predicted in function of their *likelihood* to be collectively co-activated (by firing together) on the already existing network architecture. This likelihood of localized functional interactions on the existing neural network can be influenced by external factors such as the temporal co-occurrence of the firing activity on a certain cohort of neurons, and by other factors that are intrinsic to the network architecture such as, among the most important, the *network topology*.

**Fig. 5:**
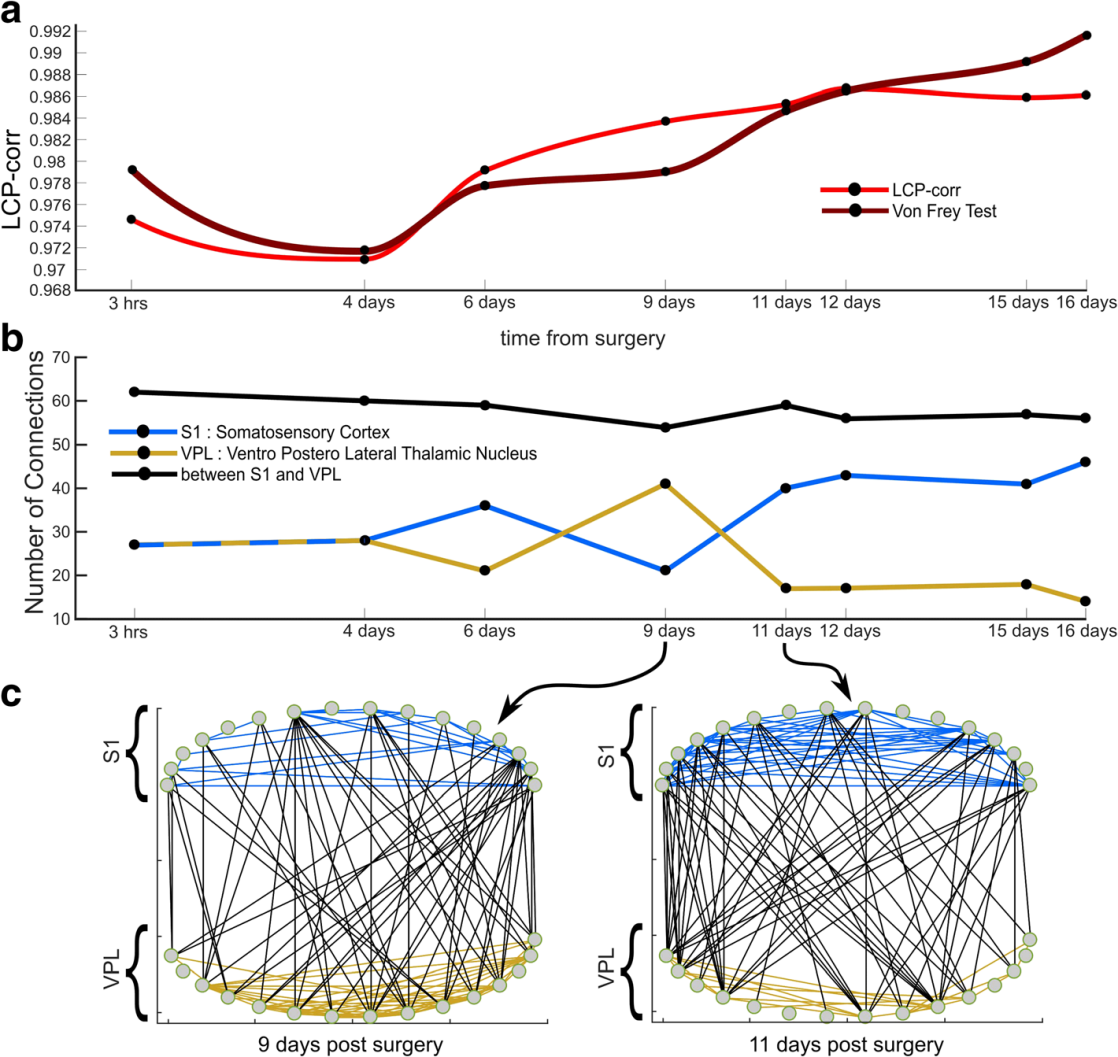
**a** Time evolution for the measure of Von Frey test superimposed to the LCP-corr. LCP-corr shows a high correlation (Pearson = 0.91, Spearman = 0.95) with the Von Frey test, suggesting that the behavioral response to pain during consecutive time points is in high correspondence with the changes of LCP-corr in the functional connectome of the rat brain. Since the curves have been stretched in order to fit in the subplot and this could misleadingly suggest a linear trend, a scale-invariant plot is provided for clarity in Additional file 1: Figure S2 (**b**) The plot highlights how the number of connections changes in time between S1 and VPL (*black*, inter regional), within S1 (*blue*, intra-regional) and within VPL (*Ochre*, intra-regional). The main variation of LCP-corr is between 4 and 11 days whereas is more stable before 4 days and after 11 days. This increase of the measure implies that significant modifications of the connectivity are occurring at the local community level. This is evident from the higher variability in the number of intra-regional links in the same interval, between 4 and 11 days. In comparison, the number of inter-regional connections between S1 and VPL is more stable, suggesting that the chronic pain produces more activity at local scale rather than global scale. **c** Representation of the brain network at two time points: 9 days and 11 days. The number of intra S1 links (*blue*) increased whereas the intra VPL links (*ochre*) decreased from 9 to 11 days. Instead, the number of links between S1-VPL remained almost the same

In 2013, Cannistraci et al. noticed that considering only the network topology, the second interpretation of the Hebbian learning could be formalized as a mere problem of topological link prediction in complex networks. The rationale is the following. The network topology plays a crucial role in isolating cohorts of neurons in functional communities that naturally and preferentially - by virtue of this predetermined local-community topological organization - can perform local processing. In practice, the local-community organization of the network topology creates a physical and structural ‘energy barrier’ that allows the neurons to preferentially fire together within a certain community and therefore to add links inside that community, implementing a type of local topological learning. In few words: the local-community organization influences (by increasing) the likelihood that a cohort of neurons fires together because they are confined in the same local community, consequently also the likelihood that they will create new connections inside the community is increased by the mere structure of the network topology. Inspired by this intuition, Cannistraci et al. called this local topological learning theory *epitopological learning*, which stems from the second interpretation of the Hebbian learning. The definition was not clearly given in the first article (Cannistraci et al. 2013a) that was immature, and therefore we now provide an elucidation of the concepts behind this theory by offering new definitions. *Epitopological learning* occurs when cohorts of neurons tend to be preferentially co-activated because they are topologically restricted in a local community, and therefore they tend to facilitate learning of new network features by forming new connections instead of merely retuning the weights of existing connections. As a key intuition, Cannistraci et al. postulated also that the identification of this form of learning in neuronal networks was only a special case; hence the *epitopological learning* and the associated *local-community-paradigm (LCP)* were proposed as local rules of learning, organization and link-growth valid in general for topological link prediction in any complex network with LCP architecture (Cannistraci et al. 2013a). On the basis of these ideas, they proposed a new class of link predictors that demonstrated - also in following studies of other authors - to outperform many state-of-the-art local-based link predictors (Cannistraci et al. 2013a; Liu et al. 2013; Tan et al. 2014; Pan et al. 2016; Wang et al. 2016; Wang et al. 2016; Pech et al. 2017; Shakibian and Charkari 2017) both in brain connectomes and in other types of complex networks (such as social, biological, economical, etc.). In addition, they proposed that the LCP is a necessary paradigm of network organization to trigger epitopological learning in any type of complex network, and that LCP-corr is a measure to quantitatively evaluate the extent to which a given complex network is organized according to the LCP. In conclusion, the LCP originated from the initial idea to explain how the network topology indirectly influences the process of learning a memory by adding new connections in a network of neurons, therefore it makes sense that is a good marker (Fig. 5a) for detecting the rewiring correlates of pain.

**Fig. 6:**
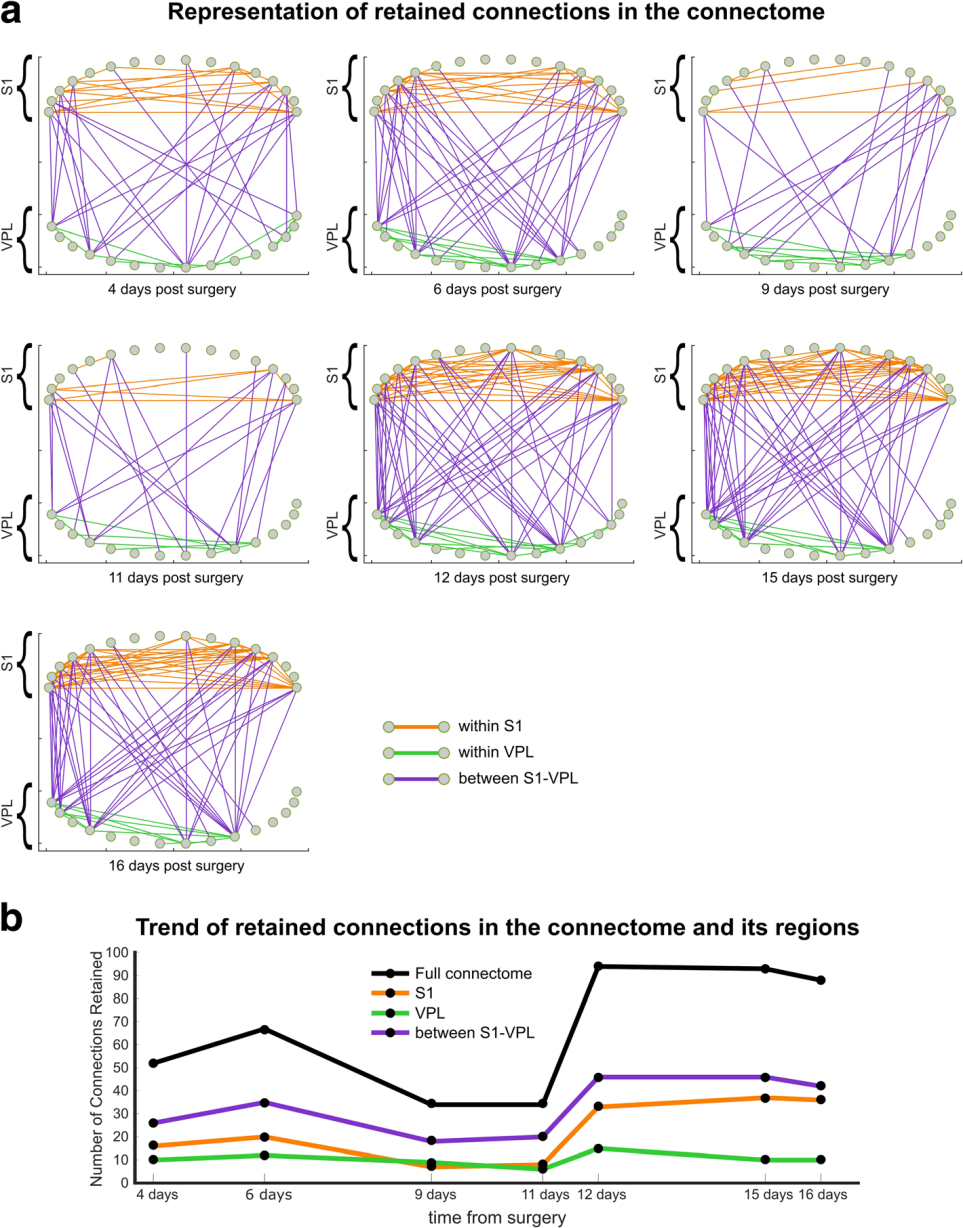
**a** Network representation of the connections retained between consecutive time steps, within S1 (*orange*), within VPL (*green*) and between S1-VPL (*purple*). E.g. 3 h/4d point represents the connections retained from time step of 3 h to 4 days post surgery. The density of the links is representative of the stability of the connectivity in the time interval. A lower density is noticed from 3 h/4d to 9d/11d and in particular at 6d/9d and 9d/11d, in fact the central intervals 4d/6d – 6d/9d and 6d/9d - 9d/11d are the ones that exhibit the highest change in the number of intra-regional links as highlighted in Fig. 5b. Instead, the final time intervals (from 11d/12d to 15d/16d) show higher density and therefore greater stability. **b** Plot of the number of retained connections within the full connectome, within S1, within VPL and between S1-VPL. Retained links represent maintained communication. A permanent engram associated with chronic pain memory seems to appear and maintain its architecture in the entire S1-VPL region starting from the 11th day

As a matter of fact, if we consider - like we did in Fig. 5b-c - the connectome that at each time step has the LCP-corr value closer to the average LCP-corr value in that specific time step, we obtain a representative network organization that should be associated to pain according to the LCP-corr measure. Indeed, in Fig. 5b we discover that the number of connections between S1 and VPL (black line) remains stable over time steps, while the number of connections in S1 (blue line) is remarkable and stably higher than in VPL (yellow line) starting from 11 days forwards. This stable depletion of connections in VPL and enrichment in S1 might suggest that starting from 11 days a permanent engram associated with chronic pain memory might be formed and maintained in the S1-VPL network, and that this network arrangements correlates also with a moderate increase of LCP-corr (Fig. 5a). On the other hand, if we are interested to visualize what (and in what quantity) are the connections retained among two subsequent time steps, we need to refer to Fig. 6. Here, a period of relatively instability of the network is evident from the 4th to the 11th day where the numbers of connections retained is relatively low (Fig. 6b), while from the 11th day forward there is a period of consolidation and stability that is characterized by high numbers of retained connections. This is a confirmation that a permanent engram associated with chronic pain memory seems to appear and maintain its architecture in the entire S1-VPL region starting from the 11th day. Hence, we can conclude that the functional signal associated with pain create a network remodelling which brings to the formation of engrams (topological memory traces) associated with chronic pain. The result in Figs. 5 and 6 shows that once the realization of memory by engram’ formation is stable, it tends to remain permanent. Therefore, our findings suggest that chronic pain might be not anymore originated by an input that comes from the periphery such as in the case of acute pain. Chronic pain seems more a stable functional engram signal that is memorized and reverberates in the S1-VPL network region of the central nervous system (Zippo et al. 2015; Zippo et al. 2016). We cannot exclude that also other brain regions might be similarly affected, but in our study the S1-VPL network seems affected according to the mechanisms and dynamic we discussed.

Unexpectedly, and this is our third important finding, the power-lawness displayed a very high negative correlation (< −0.9) with the behavioural test (Fig. 3). On one side, this result is a robust confirmation that what we detected using LCP-corr as a marker for pain is replicable (as an inverse trend) at least by another marker. Yet, we remain surprised by this result and, at the moment, we are not able to advance any exhaustive discussion to motivate the topological reasons of this association between power-lawness and behavioural test. As a matter of fact, LCP-corr and power-lawness are substantially diverse topological measures and they are inspired by different theories. The LCP-corr is a deterministic measure that computes a correlation to quantify the tendency of the network to be organized in local communities, emerging from the fact that the common neighbours of each link in the network tend to cross-interact. Instead, the power-lawness is a stochastic measure that provides a *p*-value to claim whether power-law node degree distribution governs a particular network or not. Cannistraci et al. (Cannistraci et al. 2013a) already demonstrated examples of networks that although share the same identical degree distribution can have a different LCP-corr, therefore the two measures seems not formally and theoretical correlated. In addition, the use of power-lawness we propose in our study is innovative and inedited. As we said above, and we stress here, power-lawness is generally used as a statistical test for checking whether a network follows (or not) a power law distribution. If its *p*-value is lower than or equal to 0.1 (attention: this threshold is not 0.05 or 0.01, which are the one usually used in statistics, for details refer to the original article by Clauset et al. (Clauset et al. 2009) then the network does not follow node power law distribution. It is known that brain functional networks are not power law, and this is also confirmed by our results in Fig. 3 where the *p*-values of power-lawness over the time steps are always significantly lower than the 0.1 threshold. However, to the best of our knowledge, nobody in previous literature used this test as a topological marker for network dynamics and our innovative exploitation of this network statistics triggers a new discovery on its intrinsic behaviour. The formal and theoretical basis behind this last result will require further investigations by means of dedicated studies in the future.

In normal conditions, brain connectomes follow LCP organization (Cannistraci et al. 2013a) and do not show power-law distribution (Gastner and Ódor 2016). Interestingly, our results suggest that at the time of pain peak (acute pain at 4 days after surgery) the local community organization of the network is impaired (LCP-corr value decreases) and simultaneously the network power-lawness is significantly increased. Therefore, the two topological markers point out a significant deviation from the state of normality. Whereas, during pain chronicisation (from 11 days after surgery) the network tends towards higher level of LCP organization and, simultaneously, the power-lawness tends to significantly decrease (Fig. 3). Therefore, both LCP and power-lawness suggest that at the time of acute pain, there is an enrichment of neural connections firing that suddenly makes the functional connectome less LCP and more power-law. Then, eventually, during chronic pain phase the network recovers a more *community-oriented* organization, and connections gradually and cumulatively establish a stable memory of pain, which is carved in the network topology by engram formation.

Finally, although our study suffers the limitation of an experiment based on one single rat, we stress that many of our findings meet confirmations and are in agreement with the previous literature. Nevertheless, we think that our encouraging results should be taken with a ‘pinch of salt’, and that further topological analysis of the rewiring correlates of pain in the brain time-varying functional connectomes of rat models should be performed to dig deeply into the neurobiological aspects of our discovery. In conclusion, the complex network topological analysis here proposed and the consequent results are promising achievements in the understanding of network-based mechanisms of learning, memory and engram formation, by exploiting abstract network representations of real brain connectomes. In particular, this study offers a proof of concept that advocates the designing and investigation of markers for objective pain quantification based on topological network analysis of functional brain connectome rewiring.

## Additional file

Additional file 1: Supplementary information. (DOCX 391 kb)
