# Supplementary material for: Can local-community-paradigm and epitopological learning enhance our understanding of how local brain connectivity is able to process, learn and memorize chronic pain?
Source: Appl Netw Sci. 2017 Aug 30;2(1):28. doi: 10.1007/s41109-017-0048-x (PMC6214247; doi:10.1007/s41109-017-0048-x)
Supplement: Supplementary file 1 — Supplementary information. (DOCX 391 kb) [file 41109_2017_48_MOESM1_ESM.docx]

**Additional file**

|  | Max correlation | Max p-value | Pearson correlation | Spearman correlation | Pearson p-value G | Spearman p-value G | Pearson p-value DP | Spearman p-value DP |
| --- | --- | --- | --- | --- | --- | --- | --- | --- |
| LCP-corr | 0.939 | **0.006** | 0.934 | 0.939 | 0.002 | 0.003 | 0.004 | 0.006 |
| Power-law | -0.936 | **0.002** | -0.936 | -0.909 | 0.001 | 0.002 | 0.002 | 0.003 |
| Char Path | -0.928 | **0.006** | -0.899 | -0.928 | 0.003 | 0.005 | 0.005 | 0.006 |
| EBC | -0.927 | **0.006** | -0.897 | -0.927 | 0.003 | 0.005 | 0.005 | 0.006 |
| SW-omega | 0.893 | **0.007** | 0.893 | 0.864 | 0.003 | 0.002 | 0.007 | 0.002 |
| Closeness | -0.842 | **0.021** | -0.807 | -0.842 | 0.019 | 0.021 | 0.007 | 0.009 |
| Modularity | -0.803 | **0.033** | -0.802 | -0.803 | 0.026 | 0.026 | 0.037 | 0.033 |
| Efficiency | -0.770 | **0.050** | -0.705 | -0.770 | 0.055 | 0.050 | 0.050 | 0.041 |
| BC | -0.758 | 0.113 | -0.747 | -0.758 | 0.072 | 0.112 | 0.071 | 0.113 |
| Clustering | -0.347 | 0.448 | -0.276 | -0.347 | 0.396 | 0.428 | 0.396 | 0.448 |
| Radiality | 0.339 | 0.526 | 0.276 | 0.339 | 0.505 | 0.513 | 0.513 | 0.526 |
| Struc Cons | -0.252 | 0.552 | -0.168 | -0.252 | 0.531 | 0.552 | 0.507 | 0.537 |
| SW-sigma | -0.111 | 0.848 | -0.110 | -0.111 | 0.866 | 0.848 | 0.855 | 0.827 |
| Avg Deg | 0.103 | 0.884 | 0.103 | 0.031 | 0.863 | 0.875 | 0.884 | 0.890 |

**Additional file 1: Table S1.** The table provides the results for the correlation analysis of the topological network measures with the behavioural test and for the related statistical test, analogously to Table 2 in the main article. However, in this case, while the topological measures are interpolated using the PCHIP method, the real behavioural signal (Von Frey test along time) and the related null-models are reconstructed using the spline interpolation procedure.

|  | Max correlation | Max p-value | Pearson correlation | Spearman correlation | Pearson p-value D | Spearman p-value D | Pearson p-value GP | Spearman p-value GP |
| --- | --- | --- | --- | --- | --- | --- | --- | --- |
| Power-law | -0.947 | **0.005** | -0.944 | -0.947 | 0.001 | 0.004 | 0.004 | 0.005 |
| LCP-corr | 0.933 | **0.024** | 0.873 | 0.933 | 0.022 | 0.024 | 0.027 | 0.020 |
| Closeness | -0.922 | **0.011** | -0.901 | -0.922 | 0.008 | 0.011 | 0.007 | 0.009 |
| SW-omega | 0.891 | **0.014** | 0.891 | 0.802 | 0.012 | 0.011 | 0.014 | 0.007 |
| Efficiency | -0.871 | **0.029** | -0.837 | -0.871 | 0.027 | 0.029 | 0.029 | 0.028 |
| Char Path | -0.867 | **0.024** | -0.864 | -0.867 | 0.021 | 0.024 | 0.020 | 0.017 |
| EBC | -0.866 | **0.025** | -0.860 | -0.866 | 0.023 | 0.025 | 0.020 | 0.018 |
| Modularity | -0.742 | 0.121 | -0.695 | -0.742 | 0.123 | 0.121 | 0.132 | 0.114 |
| BC | -0.640 | 0.140 | -0.640 | -0.634 | 0.140 | 0.149 | 0.137 | 0.146 |
| Radiality | 0.519 | 0.281 | 0.461 | 0.519 | 0.280 | 0.269 | 0.290 | 0.281 |
| Clustering | -0.448 | 0.448 | -0.329 | -0.448 | 0.439 | 0.436 | 0.441 | 0.448 |
| Struc Cons | -0.239 | 0.622 | -0.182 | -0.239 | 0.636 | 0.622 | 0.613 | 0.609 |
| Avg Deg | -0.162 | 0.845 | -0.075 | -0.162 | 0.846 | 0.845 | 0.840 | 0.841 |
| SW-sigma | 0.090 | 0.859 | 0.090 | 0.019 | 0.852 | 0.849 | 0.859 | 0.864 |

**Additional file 1: Table S2.** The table provides the results for the correlation analysis of the topological network measures with the behavioural test and for the related statistical test, analogously to Table 2 in the main article. However, in this case, while the topological measures are interpolated using the PCHIP method, the real behavioural signal (Von Frey test along time) and the related null-models are reconstructed using the linear interpolation procedure.


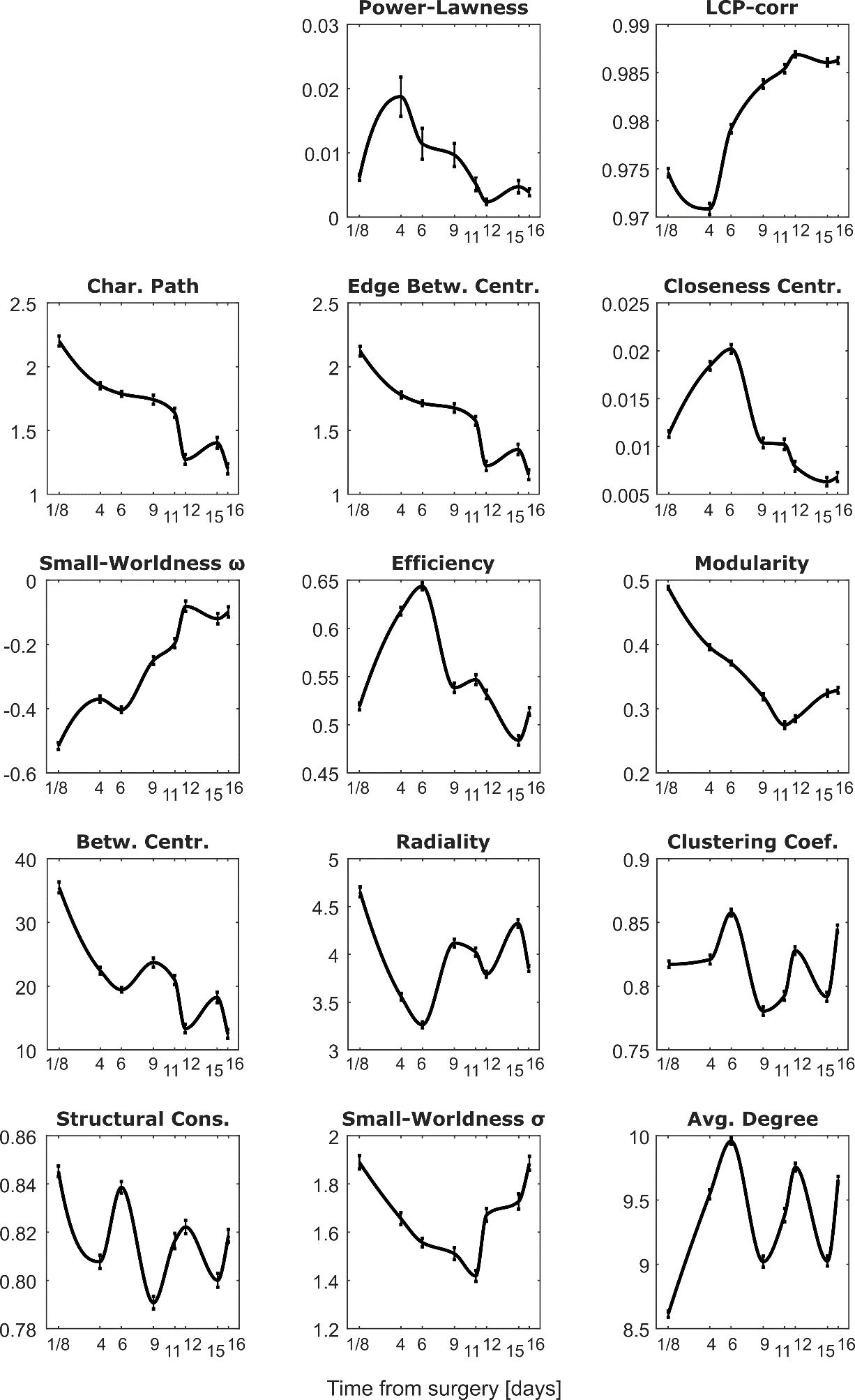


**Additional file 1: Figure S1.** The figure shows the evolution in time for the topological measure curves. In correspondence to the time points in which the data were recorded, the error bars are reported. They represent an error interval centered on the mean and wide twice the standard error, computed over the 400 values of the topological measure evaluated on the set of 400 short-time-varying connectomes considered for each time step. The figure highlights that the standard error is generally low enough to consider the uncertainty on the mean value negligible. Therefore, we can adopt the unique mean value as a reliable estimate to reconstruct the overall signal with an appropriate accuracy, and the data reliability is the main assumption for a correct usage of the interpolation procedure. The only exception is the standard error of the power-lawness for the time steps 4, 6 and 9 days. However, the error interval corresponding to the peak of acute pain (day 4) does not overlap with the following ones (days 6-9), therefore the main trend should not be affected. Note that the curves have been obtained using PCHIP interpolation on the original 8 time points and are equivalent to the ones shown in Fig. 3.


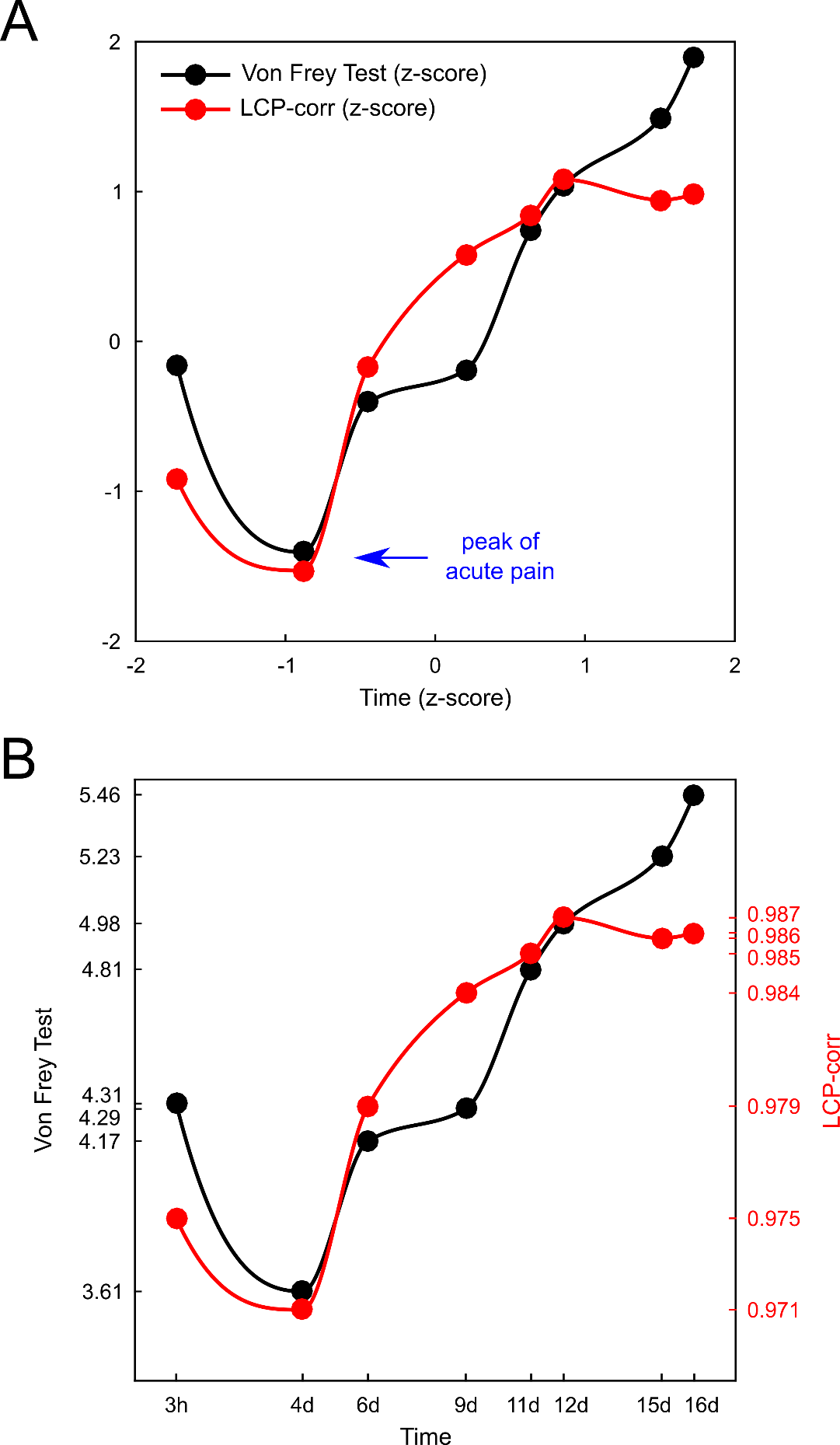


**Additional file 1: Figure S2.** Comparison of the Von Frey test and LCP-corr curves over time in a scale-invariant representation. Both the interpolated curves and the time steps have been normalized using the z-score and the values have been plotted. For each curve, the 8 dots correspond to the original time points in which the data have been acquired. The two subplots differ only on the axes labels: (A) indicates the z-scored values whereas (B) the original values. From the plots it is evident that the curves follow a similar nonlinear trend, with an initial descent toward their minimum value that corresponds to the peak of acute pain.


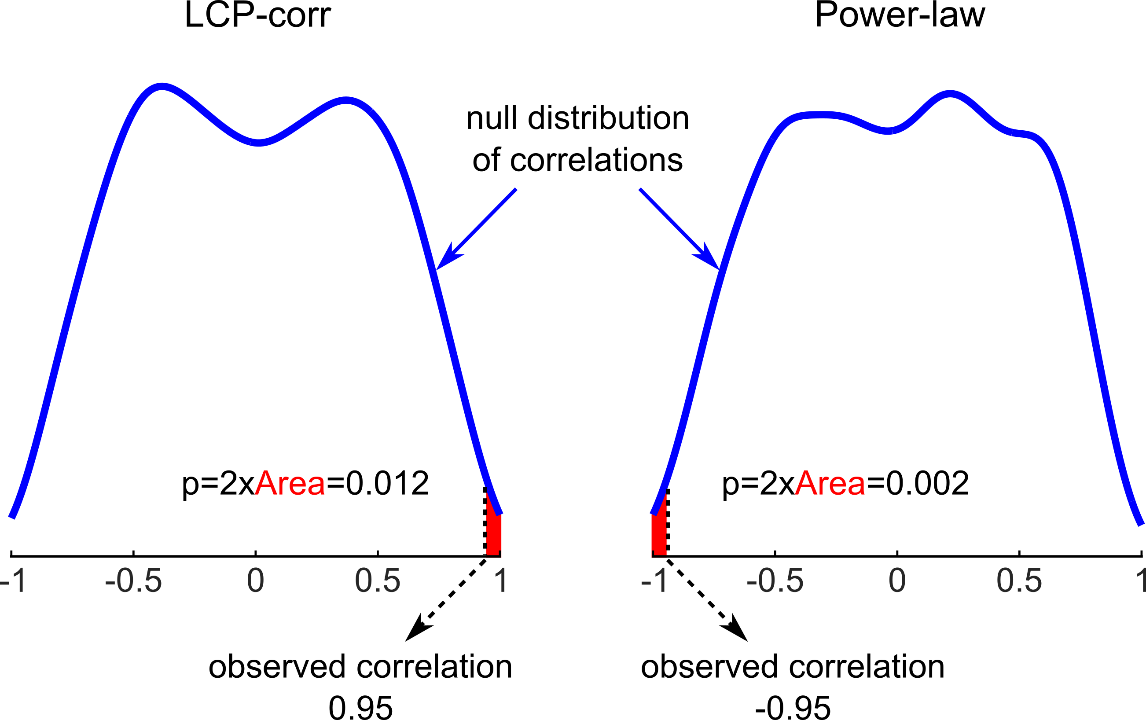


**Additional file 1: Figure S3.** Statistical test for the correlation of the topological measures LCP-corr and Power-law with the Von Frey behavioural signal. The null distribution of correlations is plotted in blue (non-parametric estimation from 10000 values using the MATLAB function *ksdensity*). The observed correlation between the topological measure and the behavioural signal is indicated using a black arrow. The area corresponding to the values equal or more extreme than the observed correlation is highlighted in red. The p-value of the two-tailed test is reported. Note that for LCP-corr the results correspond to the usage of the Spearman correlation and the null-model Gaussian white noise (G), whereas for Power-law they are related to the Pearson correlation and the null-model distribution-preserving (DP).
